# Simultaneous assessment of blood flow and myelin content in the brain white matter with dynamic [11 C]PiB PET: a test-retest study in healthy controls

**DOI:** 10.1186/s13550-024-01107-4

**Published:** 2024-05-27

**Authors:** Arya Yazdan-Panah, Benedetta Bodini, Théodore Soulier, Mattia Veronese, Michel Bottlaender, Matteo Tonietto, Bruno Stankoff

**Affiliations:** 1 Sorbonne Université, Institut du Cerveau - Paris Brain Institute - ICM, CNRS, Inria, AP-HP, Hôpital de la Pitié Salpêtrière, Paris, F-75013 Inserm France; 2 Sorbonne Université, Institut du Cerveau - Paris Brain Institute -, ICM, CNRS, Inserm, AP-HP, Hôpital de la Pitié Salpêtrière, Paris, F-75013 France; 3 Department of Information Engineering (DEI), University of Padua, Padua, Italy; 4 Department of Neuroimaging, Institute of Psychiatry, Psychology & Neuroscience, King’s College London, London, UK; 5 Université Paris-Saclay, CEA, CNRS, Inserm, BioMaps, Service Hospitalier Frédéric Joliot, Orsay, France; 6 Roche Pharma Research and Early Development, Biomarkers & Translational Technologies, Roche Innovation Center Basel, Basel, Switzerland

**Keywords:** Cerebral blood flow, Myelin, PET, ^11^C-PIB, White matter, Multiple sclerosis

## Abstract

**Background:**

Exploring the relationship between oxygen supply and myelin damage would benefit from a simultaneous quantification of myelin and cerebral blood flow (CBF) in the brain’s white matter (WM). To validate an analytical method for quantifying both CBF and myelin content in the WM using dynamic [^11^C]PiB positron emission tomography (PET).

**Methods:**

A test-retest study was performed on eight healthy subjects who underwent two consecutive dynamic [11 C]PiB-PET scans. Three quantitative approaches were compared: simplified reference tissue model 2 (SRTM2), LOGAN graphical model, and standardized uptake value ratio (SUVR). The sensitivity of methods to the size of the region of interest was explored by simulating lesion masks obtained from 36 subjects with multiple sclerosis. Reproducibility was assessed using the relative difference and interclass correlation coefficient. Repeated measures correlations were used to test for cross-correlations between metrics.

**Results:**

Among the CBF measures, the relative delivery (R1) of the simplified reference tissue model 2 (SRTM2) displayed the best reproducibility in the white matter, with a strong influence of the size of regions analyzed, the test-retest variability being below 10% for regions above 68 mm^3^ in the supratentorial white matter. [^11^C]PiB PET-derived proxies of CBF demonstrated lower perfusion of white matter compared to grey matter with an overall ratio equal to 1.71 ± 0.09 when the SRTM2-R1 was employed. Tissue binding in the white matter was well estimated by the Logan graphical model through estimation of the distribution volume ratio (LOGAN-DVR) and SRTM2 distribution volume ratio (SRTM2-DVR), with test-retest variability being below 10% for regions exceeding 106 mm^3^ for LOGAN-DVR and 300 mm^3^ for SRTM2-DVR. SRTM2-DVR provided a better contrast between white matter and grey matter. The interhemispheric variability was also dependent on the size of the region analyzed, being below 10% for regions above 103 mm^3^ for SRTM2-R1 and above 110 mm^3^ for LOGAN-DVR. Whereas the 1 to 8-minute standardized uptake value ratio (SUVR1-8) showed an intermediary reproducibility for CBF assessment, SUVR0-2 for perfusion or SUVR50-70 for tissue binding showed poor reproducibility and correlated only mildly with SRTM2-R1 and LOGAN-DVR estimations respectively.

**Conclusions:**

[^11^C]PiB PET imaging can simultaneously quantify perfusion and myelin content in WM diseases associated with focal lesions. For longitudinal studies, SRTM2-R1 and DVR should be preferred over SUVR for the assessment of regional CBF and myelin content, respectively.

**Trial registration:**

European Union Clinical Trials Register EUDRACT; EudraCT Number: 2008-004174-40; Date: 2009-03-06; https//www.clinicaltrialsregister.eu; number 2008-004174-40.

**Supplementary Information:**

The online version contains supplementary material available at 10.1186/s13550-024-01107-4.

## Introduction

The brain is the organ with the greatest metabolic demand in humans, accounting for more than 20% of the body’s energy consumption while representing only 2% of the body mass [1]. The brain’s energy expenditure is mainly made through oxidative phosphorylation and aerobic glycolysis, which require a constant delivery of oxygen and glucose that can be regulated through the modulation of cerebral blood flow (CBF). Such energetic requirements are not homogeneously distributed and show large regional variations, the gray matter consuming twice as much oxygen as the white matter (WM) in the mature brain [2].

As regional CBF regulation is coupled to metabolic expenditure, a range of imaging methods has been proposed for the in vivo quantification of brain CBF [3]. While recent magnetic resonance-based techniques are gaining interest due to their non-invasive nature, their low risk and their easy implementation, the gold standard for CBF quantification remain positron emission tomography (PET) through the direct dynamic measure of oxygen radiolabeled water ([^15^O]H_2_O) [4]. PET with [^11^C]PiB, a radiotracer traditionally used to visualize in vivo cortical amyloid-β depositions in Alzheimer’s disease [5], has been applied for the estimation of CBF in the grey matter [6] and [^11^C]PiB-derived proxies of CBF were shown to correlate with [^15^O]H_2_O PET perfusion measures in cortical regions. This novel application of [^11^C]PiB PET opened the perspective of the simultaneous measure of amyloid deposition and CBF through a single dynamic [^11^C]PiB PET exam which has subsequently been applied to detect perfusion changes in patients with Alzheimer’s disease [7, 8]. Good reproducibility of [^11^C]PiB PET-derived estimations of CBF was further demonstrated in the grey matter (GM) of cognitively unimpaired, mildly impaired, and with Alzheimer’s disease dementia [9].

Amyloid PET tracers, including [^11^C]PiB, have also been applied as markers of myelin content in the WM, due to a similar conformation of β-sheet proteins localized in amyloid plaques and myelin [10–13]. Their use allows to capture the dynamics of demyelination and remyelination in focal demyelinating lesions in diseases such as multiple sclerosis (MS) [14–16]. Such MS lesions have a typical perivenular distribution and it was suggested that perfusion changes may impact their formation and fate as they are characterized by a drastic and heterogeneous decrease in CBF [17, 18], tend to be more demyelinated and more persistent in areas of low oxygenation [19–22], whereas their appearance may be preceded by a transient increase in CBF [23].

The exploration of WM pathologies could greatly benefit from an imaging modality capable of quantifying myelin content and perfusion levels simultaneously. In this paper, we compare the performance of complementary analytical alternatives to quantify these two parameters using dynamic [^11^C]PiB brain PET imaging. Specifically, we tested the reproducibility, and sensitivity to size of the region of interest (ROI) for quantitative parameters derived from the Simplified Reference Tissue Model, Logan Graphical methods, and SUVR metrics. Given the good performance of [^11^C]PiB to quantify beta-amyloid aggregate and perfusion in GM [9], we expected the tracer to perform equally well in WM tissue.

## Materials and methods

### Dataset and study design

Eight healthy individuals (5 women, age: 31 ± 6 years) with no previous history of neurological disorder were included in this study. Each participant underwent an MRI and a [^11^C]PiB PET dynamic PET scan at study entry and repeated the [^11^C]PiB PET dynamic PET scan 57 ± 27 days later.

We also included 36 subjects with MS (21 women, age: 48 ± 11 years), who were diagnosed according to the 2005 revised McDonald criteria. These subjects allowed us to define masks of white matter lesions that reflected real MS lesions, both in shape, size, and brain location, and that could be used to study the effect of ROI size on the reproducibility of the metrics of interest.

### Data acquisition

#### PET

All dynamic PET images were acquired on a high-resolution research tomograph (HRRT) (CPS Innovations, Knoxville, TN, USA). Briefly, the 90-minute acquisition was started coincidentally with a 1-minute intravenous bolus injection of [^11^C]PiB (injected activity: 358 ± 34MBq).

Images were reconstructed using a three-dimensional ordinary Poisson-ordered subset expectation maximization algorithm with 10 iterations, with smoothing, with software developed by the HRRT users community [24]. To reduce the effect of partial volume in PET data, a deconvolution implementing the point spread function was applied to the reconstructed image [25]. The PET images were reconstructed on 25 time-frames (6 × 1; 6 × 2; 4 × 3; 6 × 5; 3 × 10 min), with a voxel size of 1.22 × 1.22 × 1.22 mm and a field of view 25 cm (axial) and 31.3 cm (trans-axial). Additional details can be found in [12].

### MRI

All subjects’ MRI acquisitions were performed on a 3T Siemens TRIO scanner, equipped with a 32-channel coil. The MRI protocol included a 3D T1-weighted magnetization-prepared rapid gradient-echo (MPRAGE) (echo time/inversion time/repetition time = 2.9/900/2300ms, flip angle = 8°, voxel resolution = 1 × 1 × 1.1 mm³). Patients with MS also underwent a T2-weighted (echo time/repetition time = 83/4000ms, resolution 0.9 × 0.9 × 3.0 mm³) and 3-dimensional fluid-attenuated inversion recovery (FLAIR) (echo time/repetition time = 129/8880ms, voxel resolution = 0.9 × 0.9 × 3.0 mm³).

### Data processing

#### PET quantification

PET scans were corrected for interframe subject motion using the wavelet method implemented in Piwave 7.0 [26]. The quantification of PET scans was based on the extraction of a reference region obtained with a supervised clustering algorithm [12, 27]. Briefly, the supervised clustering algorithm fits each voxel’s time activity curve (TAC) as a linear combination of kinetic classes predefined on a group of healthy subjects, using a non-negative least square estimator [27]. Kinetic classes are the standardized activity of the tracer in predefined regions of interest obtained from a reference population. The set of kinetic classes should represent all the possible ranges of tracer behavior in the brain. The kinetic classes used in this study are similar to those employed by Veronese et al. in their previous study: grey matter, white matter, and blood [12]. Only voxels located in the supratentorial GM and with a contribution of reference region type of tissue above 90% are considered as belonging to the reference region. To ensure that differences between subjects are not a result of reference region extraction we also use as a comparison point the cerebellar grey matter, widely used in the literature [28].

This reference region was employed to derive proxies of tracer binding and cerebral perfusion, using three different approaches: 1) the simplified reference tissue model 2 (SRTM2), the Logan graphical reference method (*Logan-Ref*), and standardized uptake value ratio (SUVR).

*SRTM2.* The simplified reference tissue model 2 (SRTM2) [29, 30] was used to obtain the relative delivery (R1) and the distribution volume ratio (DVR) at the voxel level. The SRTM equation is given by [30]:

$$C_T(t)=R_1 * C_{r e f}(t)+\left[k_2{ }^{\prime}-k_2\right] * C_{r e f}(t) \otimes e^{-k_2 * t}$$

where:

- ${C}_{ref}\left(t\right)$ is the TAC of the reference region
- ${C}_{T}\left(t\right)$ is the TAC of the voxel under analysis
- ${R}_{1}$ is the relative delivery ratio of the influx in the voxel under analysis to the influx in the reference region
- ${k}_{2}{\prime }$ and ${k}_{2}$ are the efflux rates in the reference region and voxel under analysis, respectively

From these parameters, the DVR (SRTM2-DVR), defined as the ratio of the total distribution volume between the target and the reference region, a representation of the binding of [^11^C]PiB, can be calculated as:

$$DVR={R}_{1}\frac{{k}_{2}{\prime }}{{k}_{2}}$$

Using basis function methods [31], it is possible to solve the equation in a time-efficient and robust way. An initial pass is performed to estimate ${R}_{1}$, ${k}_{2}^{{\prime }}$ and ${k}_{2}$, with 500 linearly spaced ${k}_{2}$ bases in [0.01;0.1] min^− 1^. Since the efflux rate from the reference region should be independent of the voxel under analysis, the ${k}_{2}^{{\prime }}$ parameter can be fixed to a common value. Fixing the ${k}_{2}^{{\prime }}$ has been shown to improve R1 estimates [32]. In this study, ${k}_{2}^{{\prime }}$ was fixed to the median value of all voxels with a DVR higher than 1.1, as recommended by Peretti et al. [33]. By doing so, the estimates of R1 significantly improve with little impact on the DVR. After fixing ${k}_{2}{\prime }$ for all brain voxels, a two-parameter fit was performed again on the first equation for the estimation of ${R}_{1}$ and ${k}_{2}$.

The *Logan-Ref* [34] was applied at the voxel level to obtain another estimation of the DVR (LOGAN-DVR). *Logan-Ref*, applied to [^11^C]PiB dynamic imaging has been demonstrated to provide a reliable [12] estimation of myelin content in the white matter [10, 11, 13]. The ${k}_{2}{\prime }$ estimation was ignored during the Logan-Ref estimation as it was shown by Veronese et al. [12] that estimating the Logan-Ref with and without k2 provided similar results. The start time for the line fit was set at 30 min, i.e., once the linear phase of the Logan plot was reached. No proxy of CBF was derived from Logan graphical analysis.

The Standardized Uptake Value Ratio (SUVR) consists of a time-averaged ratio of the signal over the signal in the reference region. Briefly, each frame of the dynamic acquisition is normalized by the signal in the reference region at the same frame. SUVR frames are then averaged in the wanted time frames. Please note that while SRTM2 and LOGAN require full dynamic scanning while SUVR estimates only use a particular time window. The early frames 0–2 min and 1–8 min (SUVR0-2, SUVR1-8) were tested as proxies of CBF [35, 36], and the late frames, 50–70 min SUVR (SUVR50-70) as a proxy of DVR [37]. All PET modeling was written using MATLAB 2017, and the rest of the image processing pipelines were written in Python 3.7.

**Fig. 1:**
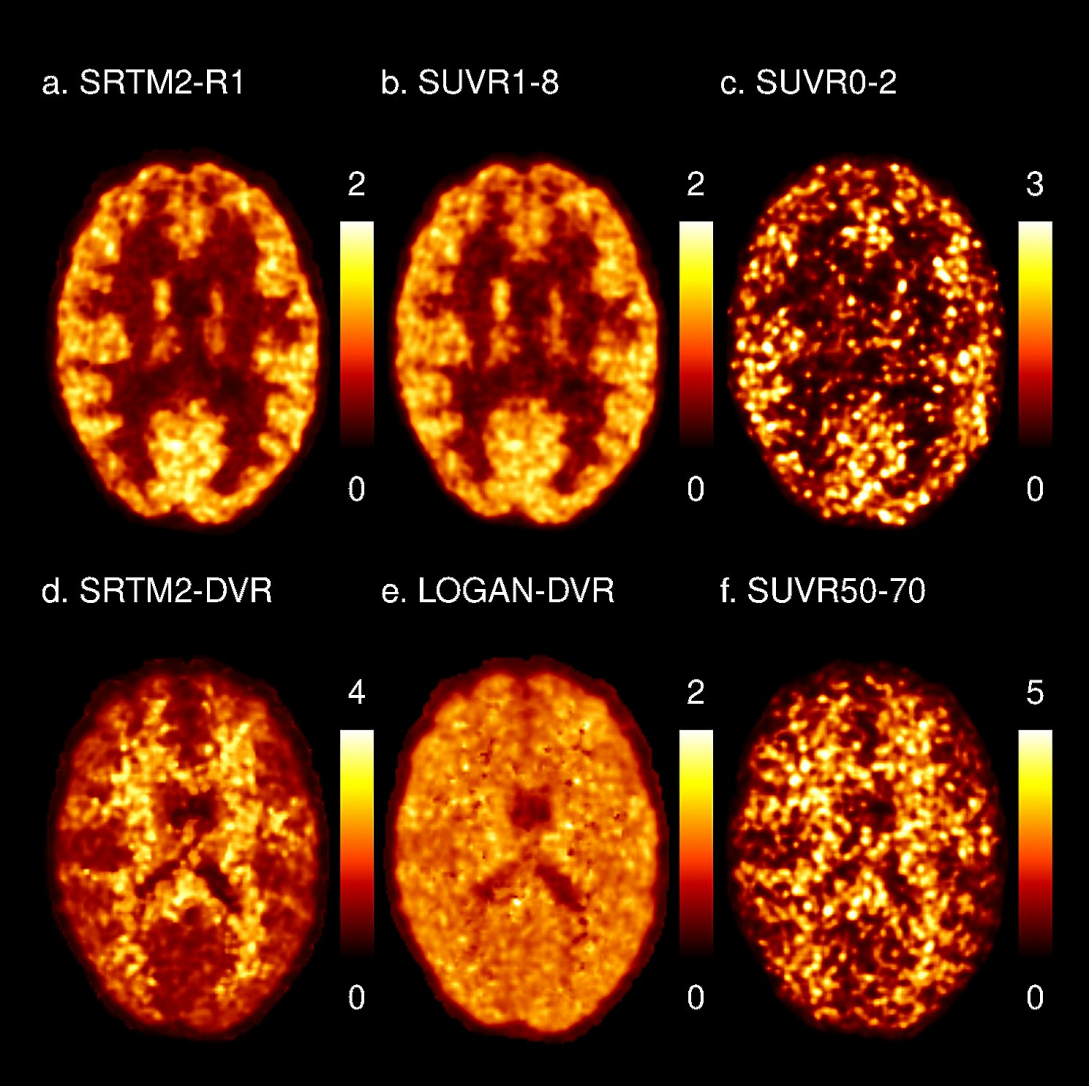
Parametric maps of perfusion and myelin content for a representative subject. The top row represents perfusion and the bottom row binding estimations. (A) SRTM2-R1: relative delivery of [^11^C]PiB as estimated by the SRTM2, (B) SUVR1-8: SUVR calculated on frames from 1 to 8 min, (C) SUVR0-2: SUVR calculated on frames from 0 to 2 min, (D) DVR-SRTM2: DVR as estimated by the SRTM2, (E) LOGAN-DVR: DVR as estimated by the Logan graphical reference method, (F) SUVR50-70: SUVR calculated on frames from 50 to 70 min

Examples of all parametric maps obtained from a representative subject are shown in Fig. 1.

### MRI processing

3D-T1 images were corrected for field bias in all subjects using the N4 algorithm [38] implemented in Advanced Normalization Tools (http://stnava.github.io/ANTs/) (ANTs). 3D-T1s of healthy controls were processed using FreeSurfer 6.0.0 standard pipeline. Pial surfaces and grey/white interfaces were manually corrected by a neurologist when necessary. The supratentorial and cerebellar white matter were extracted from the FreeSurfer processing. The supratentorial WM was then subdivided into the following areas: cingular, frontal, insular, occipital, parietal, and temporal regions. For comparative purposes, GM was also considered in the supratentorial and cerebellar GM.

In subjects with MS, lesions were manually contoured on the T2-weighted MRI with the FLAIR as a reference by a senior neurologist and registered onto the 3D-T1.

### Standard space processing

Time-averaged PET images were obtained from the motion-corrected PET image and rigidly aligned to the subject’s corresponding 3D-T1 image.

All 3D-T1 images (after lesion filling in patients [39]), were normalized to the MNI152 space using a nonlinear deformation calculated with ANTs. The same transformation was then used to bring all the previously defined ROIs (total WM and WM subregions in healthy controls and T2 lesions in patients), PET-derived metrics, and reference regions to the standard space. Once in standard space, for each region of the WM, a common mask across healthy subjects is created by intersecting the corresponding masks of each healthy individual.

T2 lesion masks of patients registered in standard space were individualized by detecting individual connected components, resulting in the generation of 2402 lesion-like ROIs with a median size of 31 mm^3^ and ranging from 1 to 74,688 mm^3^.

Aiming to assess the inter-hemispheric variability of [^11^C]PiB PET-derived metrics reflecting the tracer’s binding and cerebral perfusion, all individual lesion-like ROIs were flipped along the sagittal plane to generate contralateral lesion masks.

### Statistical analysis

Differences in SUVR, DVR, and R1 parameters between regions of interest were tested using a Student’s t-test. Correlations between metrics were assessed using a repeated measures correlation [40]. Results are reported as significant at *p* < 0.05.

For all white and grey matter regions (excluding voxels belonging to the reference region), and all lesion-like ROIs, the following two indices were employed to assess the reproducibility of the [^11^C]PiB PET-derived metrics between the first and second time-point in the 8 healthy subjects:

(i) The percentage relative difference (%RD), defined as:
  $$\% RD=200* \frac{\left|mean_{ROI}\right(Test) - mean_{ROI}(Retest\left)\right|}{mean_{ROI}\left(Test\right) + mean_{ROI}\left(Retest\right)}$$

With $mea{n}_{ROI}\left(parameter\right)$=mean value of parameter masked with ROI.

(ii) The intraclass correlation coefficient (ICC), measuring the extent of the resemblance between two observations of the same individual. The type of ICC chosen follows the recommendations of Koo and Li in their guideline for ICC selection [41]. Therefore, the model chosen is a two-way mixed effect with absolute agreement, as consistency is sought in values obtained between subjects with a single rater.
  $$ICC=\frac{M{S}_{R} - M{S}_{E}}{M{S}_{R} + (k-1) M{S}_{E} + \frac{k}{n}(M{S}_{C}-M{S}_{E})}$$

With:

- $M{S}_{R}$=mean square between different subjects;
- $M{S}_{E}$=mean square for error;
- $M{S}_{C}$=mean square between test and retest;
- $n$=number of subjects;
- $k$=number of reiterations of the experiment (test and retest).

(iii) The within-subject coefficient of variation (WSCV). As reported by Baumgartner et al. [42] this metric has relevance in the case of PET test-retest studies as it performs global scaling instead of scaling at the individual level, therefore allowing to retain outliers if they exist.

SRTM2-R1 quantified using the SVCA and the cerebellar grey matter as reference regions were compared using ICC, WSCV and correlated using a Pearson correlation coefficient.

Finally, to assess the inter-hemispheric variability of each metric, all lesion-like ROIs were compared to their contralateral mirror ROIs at both time points using %RD and ICC.

## Results

### Simultaneous quantification of regional CBF and myelin content using [^11^C]PiB

**Fig. 2:**
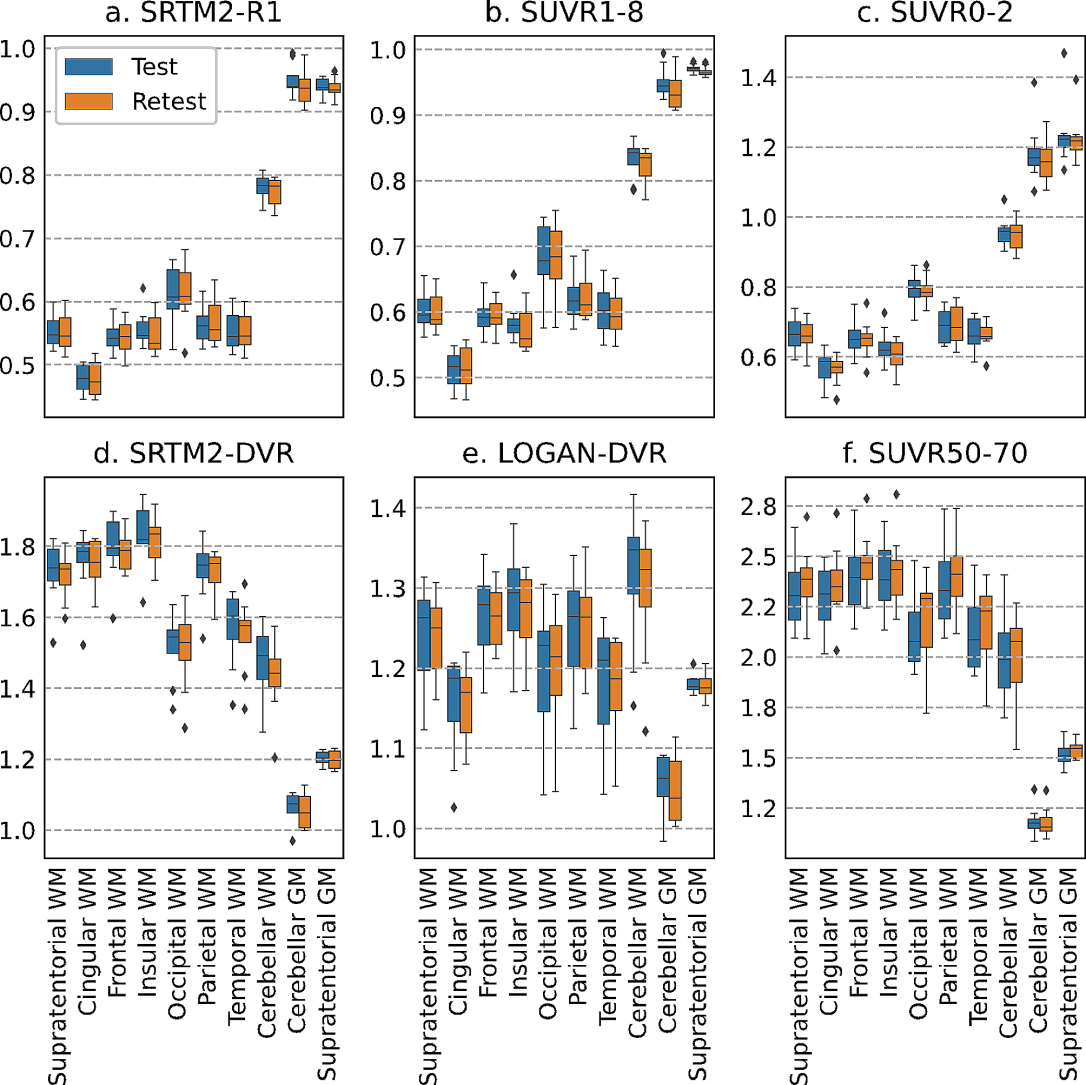
Average values of perfusion and tissue binding across brain ROIs. The top row shows perfusion and the bottom row binding estimations. From left to right, top to bottom, SRTM2-R1, SUVR1-8, SUVR0-2, SRTM2-DVR, LOGAN-DVR, SUVR50-70. Blue boxes represent the value obtained at the first visit (test), whereas orange boxes represent the values obtained at the second visit (retest). Each box’s length represents the interquartile range of the data. Whiskers are drawn within the 1.5 interquartile range. The line in the box shows the median of the data. Outside points represent potential outliers

Figure 1 presents examples of maps obtained for a representative subject in the 6 parameters studied. SRTM2-R1 estimates (proxies CBF), were significantly lower in the WM compared to the GM both at the supratentorial (WM: 0.55 ± 0.03, GM: 0.94 ± 0.01, *p* < 0.0001) and at the cerebellar level (WM: 0.78 ± 0.02, GM: 0.95 ± 0.03, *p* < 0.0001) (Fig. 2). Similar results were obtained with the other two static PET-derived cerebral perfusion proxies (SUVR0-2, SUVR1-8, Table 1). SRTM2-R1 provides a GM-WM ratio of 1.71 ± 0.09 and while having a very similar contrast compared to early frame SUVR, presents less noise compared to SUVR0-2.

**Table 1:** Observed mean PET-derived metrics

	Supratentorial WM	Supratentorial GM	*P*-value	Cerebellar WM	Cerebellar GM	*P*-value
SRTM2-R1	0.55 ± 0.03	0.94 ± 0.01	*P* < 0.001	0.78 ± 0.02	0.95 ± 0.03	*P* < 0.001
SUVR1-8	0.60 ± 0.03	0.97 ± 0.01	*P* < 0.001	0.83 ± 0.03	0.95 ± 0.02	*P* < 0.001
SUVR0-2	0.67 ± 0.05	1.24 ± 0.10	*P* < 0.001	0.96 ± 0.05	1.19 ± 0.09	*P* < 0.001
SRTM2-DVR	1.73 ± 0.09	1.20 ± 0.02	*P* < 0.001	1.47 ± 0.12	1.06 ± 0.04	*P* < 0.001
LOGAN-DVR	1.24 ± 0.07	1.18 ± 0.01	2.42E-02	1.32 ± 0.09	1.06 ± 0.04	1.06E-03
SUVR50-70	2.31 ± 0.19	1.52 ± 0.07	*P* < 0.001	2.01 ± 0.24	1.18 ± 0.08	*P* < 0.001

The average is obtained across all healthy subjects at the first time point. Results are reported as mean ± standard deviation. Significance threshold after Bonferroni correction: *p* < 4.2E-3.

On the contrary, SRTM2-DVR estimates (proxies myelin content), showed an opposite compartment variation, with WM regions displaying significantly higher values compared to GM areas both at the supratentorial (WM: 1.73 ± 0.09, GM: 1.20 ± 0.02, *p* < 0.0001) and the infratentorial level (WM: 1.47 ± 0.12, GM: 1.06 ± 0.04, *p* < 0.0001). While less pronounced, similar compartment variations are also observed with LOGAN-DVR at the supratentorial (WM: 1.24 ± 0.07, GM: 1.18 ± 0.01, *p* = 2.42E-02) and the infratentorial level (WM: 1.32 ± 0.09, GM: 1.06 ± 0.04, *p* = 1.06E-03). Both qualitatively and quantitatively in terms of GM-WM ratio, SRTM2-DVR mapping overperformed Logan-DVR. Similar contrasts were obtained with the static PET-derived binding proxy (SUVR50-70, Table 1), although the maps look noisier compared to dynamic PET quantification approaches.

### [^11^C]PiB PET-derived metrics reflecting CBF and myelin do not correlate with each other

**Fig. 3:**
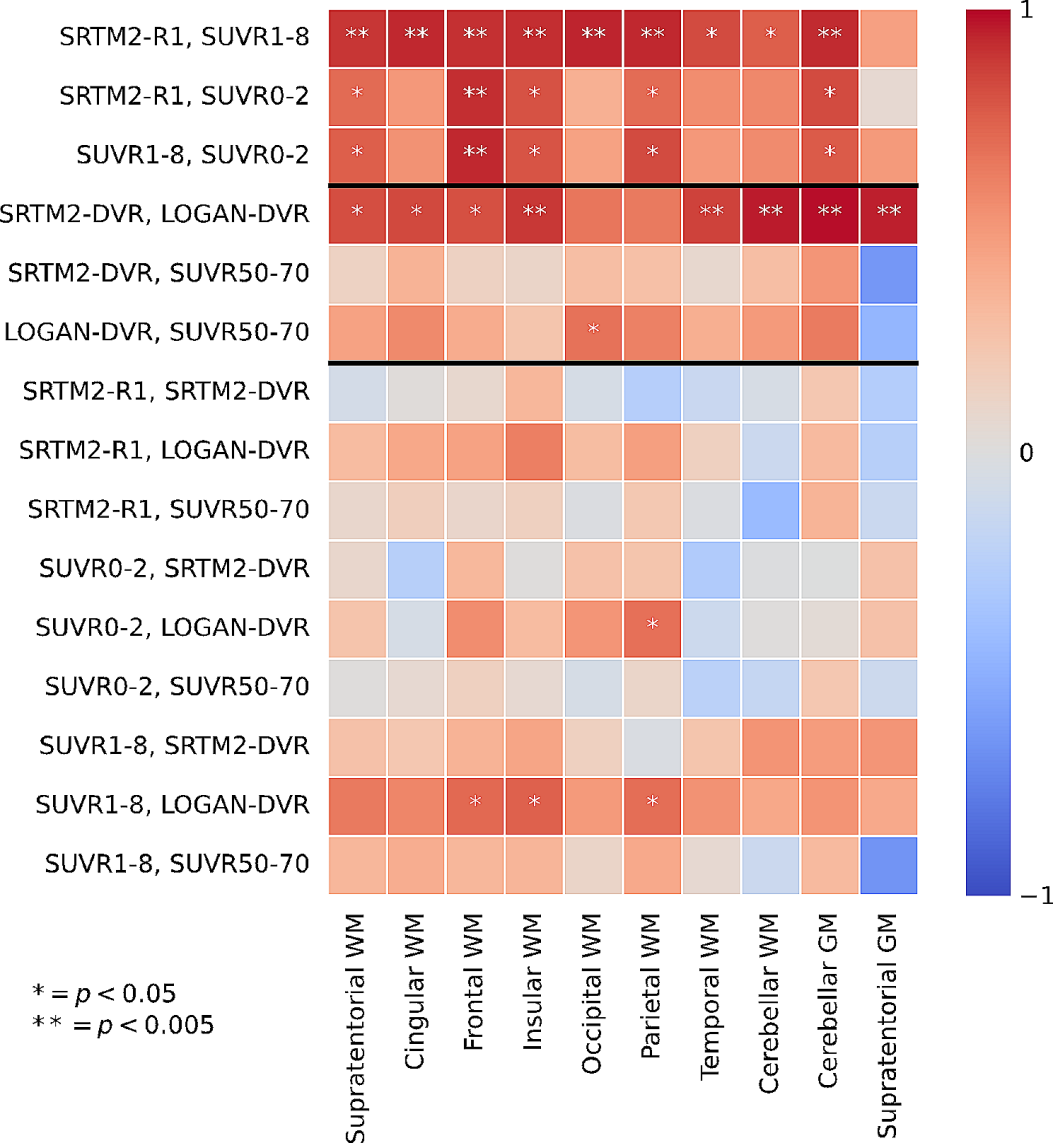
Repeated measure correlation between metrics across ROIs. Repeated measure correlations [40]. *Denotes a significance threshold of 0.05. ** Denotes significance with a threshold of 0.005, after Bonferroni correction

In all white and grey matter analyzed region, [^11^C]PiB PET-derived perfusion parameters and myelin density proxies did not significantly correlate with each other. The only exception is for SUVR1-2 and SUVR1-8 which showed some mild correlations with LOGAN-DVR that did not survive Bonferroni correction for multiple comparisons (Fig. 3).

On the contrary, WM perfusion metrics significantly correlated with each other: the strongest correlations being between SRTM2-R1 and SUVR1-8, which reached a coefficient of 0.89 in the supratentorial WM (*p* = 1.16E-03). SRTM2-R1 and SUVR1-8 also correlated well with each other in the cerebellar GM (*r* = 0.93, *p* = 3.15E-04) but did not in the supratentorial GM (*r* = 0.47 *p* = 0.21) (Fig. 3).

Similarly, SRTM2-DVR and LOGAN-DVR are parameters that correlate the best with each other, reaching a coefficient of 0.81 in the supratentorial white matter (*p* = 7.63E-03) and 0.96 in the infratentorial white matter (*p* < 0.0001) (Fig. 3). On the contrary, SUVR50-70 showed poor correlations with the other two myelin-reflecting metrics (Fig. 3).

Finally, the SRTM2-DVR and SRTM2-R1 showed the least correlations across all regions analyzed.

### Using the SVCA instead of the cerebellar grey matter as a reference region does not alter the SRTM2-R1 quantification in the white matter

When quantifying SRTM2-R1 using both the SVCA and the cerebellar grey matter reference region, we found, in the WM, a respective ICC of 0.92 and 0.90, and a respective WSCV of 1.60E-02 and 1.67E-02. In all regions both metrics were close, and one quantification did not show inflation of these results compared to the other.

In all the supratentorial white matter regions, correlations between the two quantification methods were above 0.8 and significant.

### Reproducibility of [^11^C]PIB PET-derived metrics according to brain localization

The variability of CBF proxies at the ROI level is summarized in Table 2. All the perfusion-derived metrics (SRTM2-R1, SUVR1-8, and SUVR0-2) showed similar levels of reproducibility in the supratentorial WM (SRTM2-R1: %RD = 1.74 ± 1.55, ICC = 0.92; SUVR1-8: %RD = 1.81 ± 1.51, ICC = 0.91; SUVR0-2: %RD = 1.84 ± 0.93, ICC = 0.96) and in all analyzed WM subregions, none of the methods showed variability higher than 5%.

**Table 2:** Observed test-retest variabilities for perfusion estimations

ROI Name	SRTM2-R1		SUVR1-8		SUVR0-2	
	RD [%]	ICC	RD [%]	ICC	RD [%]	ICC
Supratentorial WM	1.74 ± 1.55	0.92	1.81 ± 1.51	0.91	1.84 ± 0.93	0.96
Cingular WM	2.49 ± 1.64	0.86	2.86 ± 2.06	0.85	3.38 ± 3.59	0.82
Frontal WM	2.16 ± 1.63	0.87	2.62 ± 1.71	0.78	2.62 ± 1.85	0.94
Insular WM	2.56 ± 1.54	0.87	3.02 ± 2.17	0.80	4.61 ± 3.54	0.77
Occipital WM	1.88 ± 0.88	0.97	1.44 ± 0.91	0.98	3.32 ± 1.98	0.79
Parietal WM	2.50 ± 1.55	0.91	1.88 ± 1.56	0.94	1.72 ± 0.84	0.97
Temporal WM	2.00 ± 1.42	0.92	1.77 ± 1.52	0.94	2.86 ± 1.60	0.88
Cerebellar WM	1.92 ± 1.39	0.68	1.79 ± 0.81	0.87	1.91 ± 1.33	0.89
Cerebellar GM	1.75 ± 1.40	0.75	1.82 ± 0.91	0.78	2.11 ± 2.79	0.86
Supratentorial GM	0.77 ± 0.63	0.82	0.60 ± 0.36	0.64	1.66 ± 1.71	0.94

Results of %RD are reported as mean ± standard deviation

LOGAN-DVR and SRTM2-DVR showed very high levels of reproducibility across all investigated ROIs, with %RD consistently remaining below 5% (Table 3). By contrast, SUVR50-70 showed poor reproducibility in all WM regions analyzed, with a min %RD = 7.51 ± 4.69 (Table 3).

**Table 3:** Observed test-retest variabilities for binding parameters

ROI Name	SRTM2-DVR		LOGAN-DVR		SUVR50-70	
	RD [%]	ICC	RD [%]	ICC	RD [%]	ICC
Supratentorial WM	2.44 ± 2.38	0.78	1.98 ± 1.48	0.88	8.58 ± 5.05	0.21
Cingular WM	3.12 ± 3.52	0.63	3.16 ± 2.57	0.73	10.30 ± 5.19	-0.07
Frontal WM	2.56 ± 2.47	0.69	2.08 ± 1.57	0.82	8.56 ± 5.53	0.13
Insular WM	3.10 ± 2.33	0.67	2.28 ± 2.13	0.82	8.19 ± 5.19	0.22
Occipital WM	2.33 ± 2.42	0.92	1.99 ± 1.57	0.95	9.26 ± 3.83	0.50
Parietal WM	1.96 ± 1.74	0.87	2.03 ± 1.10	0.92	8.94 ± 5.28	0.20
Temporal WM	3.34 ± 3.14	0.83	2.22 ± 1.38	0.92	7.75 ± 5.31	0.54
Cerebellar WM	4.28 ± 3.11	0.81	2.94 ± 2.24	0.88	7.51 ± 4.69	0.75
Cerebellar GM	2.68 ± 2.09	0.73	2.19 ± 1.34	0.79	2.36 ± 1.41	0.91
Supratentorial GM	1.20 ± 0.92	0.71	0.48 ± 0.50	0.86	2.85 ± 1.84	0.62

Results of %RD are reported as mean ± standard deviation.

### At the lesion-like ROI level, the highest reproducibility is obtained with SRTM2-R1 for CBF and LOGAN-DVR for myelin content

**Fig. 4:**
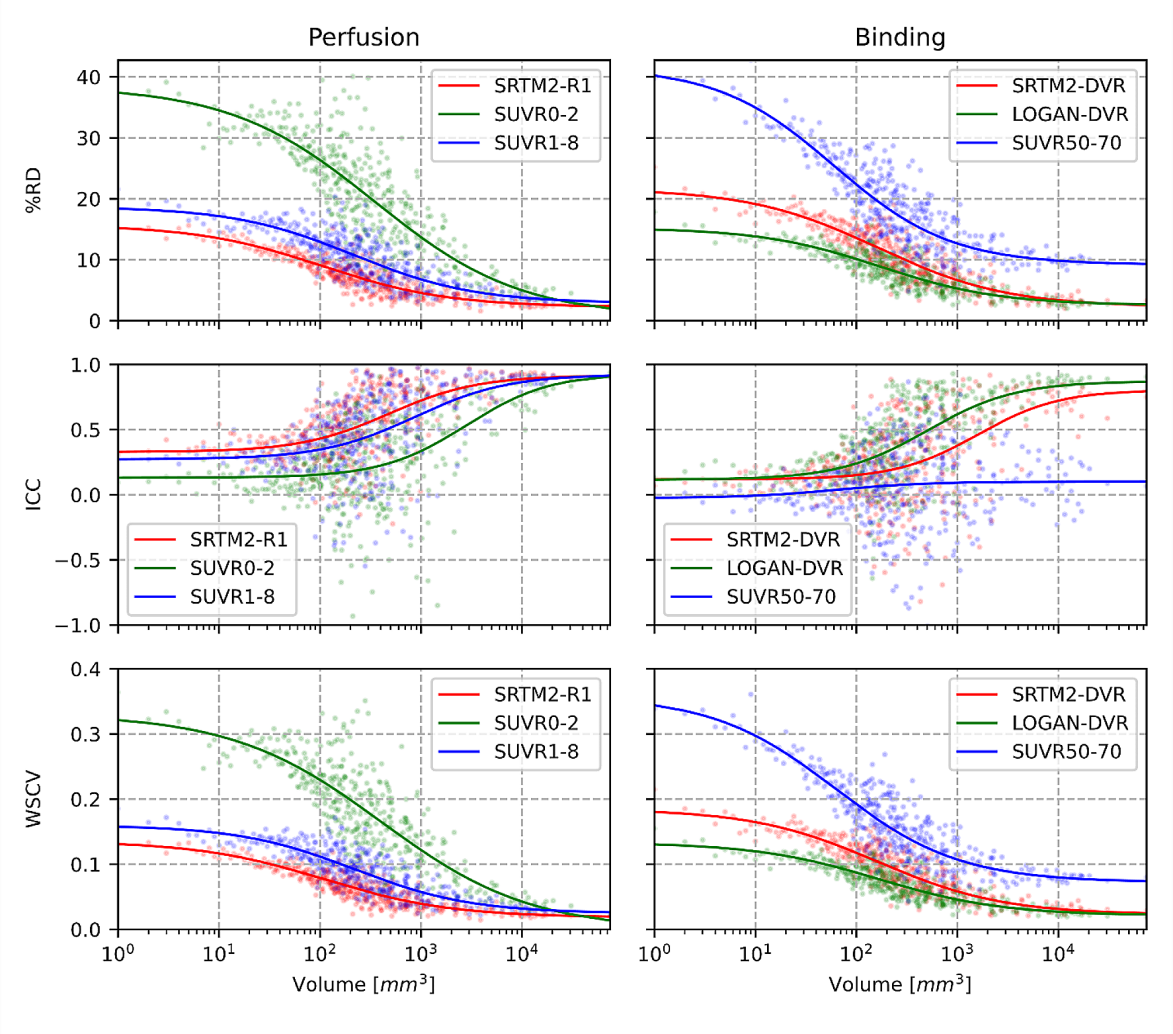
Perfusion and Binding test-retest variability/ICC/WSCV in the white matter according to ROIs volume. Mean values of each parametric map were extracted inside each lesion-like ROIs at the two-time points. Relative differences, ICCs and WSCVs were computed for all ROIs and averaged for ROIs of equal size; therefore, each point represents the mean value of %RD/ICC/WSCV at a given number of voxels. The top row shows the test-retest variability expressed in percent; the middle row presents ICCs; the bottom row presents WSCVs. On the left column, perfusion measurements are presented, with SRTM2-R1 (red), SUVR0-2 (green), and SUVR1-8(blue). On the right column, binding measures are presented, with SRTM2-DVR (red), LOGAN-DVR (green), and SUVR50-70 (blue). No transformation was applied to the data. We tested polynomial and sigmoid curves to fit the data and concluded that the sigmoid provided the best fit by using the Akaike information criteria

The test-retest variability was closely dependent upon the size of lesion-like ROIs for most analyzed metrics (Fig. 4). All %RD curves clearly decrease with the increase of ROI volume. The ICC also improved with the ROI volumes with the exception of SUVR 50–70 that did not improve in larger ROIs.

The SRTM2-R1 was the most stable measure for WM perfusion, followed by SUVR1-8. SUVR0-2 performed lower in smaller regions. With SRTM2-R1, the variability starts around 15.2% for single voxels and progressively decreases, reaching 2.38% for the biggest region considered (∼ 75 cm^3^). For all metrics, a 10% variability threshold was reached for regions above 68 mm^3^ for SRTM2-R1, 283 mm^3^ for SUVR1-8, and 2190 for SUVR0-2.

For DVR estimations, the LOGAN-DVR produced the most stable measure, showing a 14.63% variability for single voxels, which decreases to 2.76% in the biggest region (Fig. 4). A 10% variability threshold was reached for regions above 106 mm^3^ for LOGAN-DVR, 300 mm^3^ for SRTM2-DVR, and 7615 mm^3^ for SUVR50-70. SUVR50-70 showed an ICC that did not improve even in larger lesion-like ROIs.

Overall, the WSCV provided similar results compared to the %RD.

**Fig. 5:**
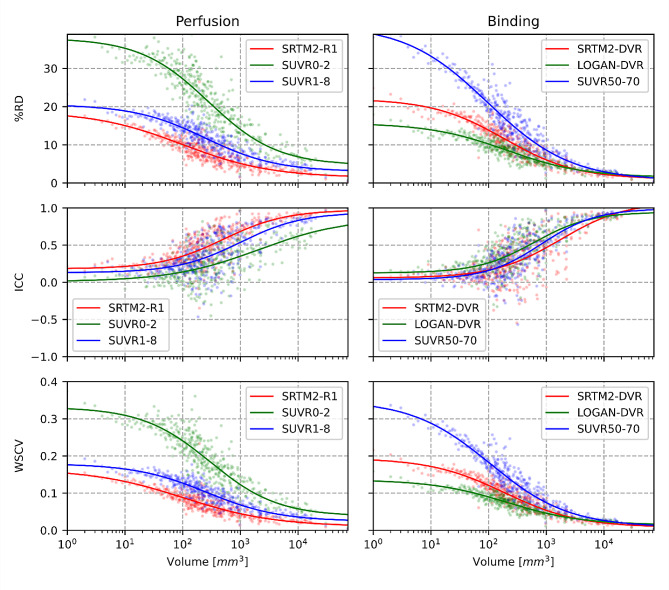
Perfusion and tissue binding inter-hemispheric variability metrics in the white matter according to ROIs volume. Mean values of each parametric map were extracted inside each lesion-like ROI and its contralateral mirror masked with the white matter. Relative differences and ICCs were computed for all ROIs and averaged for ROIs of equal size; therefore, each point represents the mean value of %RD/ICC/WSCV at a given number of voxels. The top row shows the test-retest variability expressed in percent; the middle row presents ICCs; the bottom row presents the WSCVs. On the left column, perfusion measurements are presented, with SRTM2-R1 (red), SUVR0-2 (green), and SUVR1-8(blue). On the right column, binding measures are presented, with SRTM2-DVR (red), LOGAN-DVR (green), and SUVR50-70 (blue). No transformation was applied to the data. We tested polynomial and sigmoid curves to fit the data and concluded that the sigmoid provided the best fit by using the Akaike information criteria

The interhemispheric variability was also strongly influenced by the size of the analyzed regions (Fig. 5). SRTM2-R1 and LOGAN-DVR (which are the two most reproducible metrics of CBF and tissue binding) displayed an interhemispheric variability of 16.75% and 17.93% at the voxel level respectively, which decreased down to 0.39% and 0.27% in the largest region. A 10% variability was reached in regions above 103 mm^3^ with SRTM2-R1, 110 mm^3^ with LOGAN-DVR, and 289 mm^3^ with SRTM2-DVR. A poor interhemispheric reproducibility was obtained with SUVR0-2, SUVR1-8, and SUVR50-70, reaching the 10% threshold at respectively 2705, 447, and 735 mm^3^. The interpretation of interhemispheric variability was not modified when WSCV was analyzed.

## Discussion

We explored here whether dynamic [^11^C]PiB PET could simultaneously quantify cerebral blood flow and myelin content in the WM with acceptable reproducibility. [^11^C]PiB PET-derived SRTM2-R1 confirmed the lower perfusion of WM compared to GM [43–46], with a ratio of 1.71 ± 0.09, in concordance with values found in the literature [43]. Among the CBF measures, the SRTM2-R1 displayed the best reproducibility in the WM, good quality parametric maps, with an influence of the size of regions analyzed on the test-retest variability. Similarly, tissue binding in the WM was well estimated by LOGAN-DVR and SRTM2-DVR, with SRTM2-DVR maps overperforming LOGAN-DVR or SUVR50-70 on data quality and contrast, and LOGAN-DVR overperforming SRTM2-DVR and SUVR50-70 in reproducibility. In the supratentorial WM, the test-retest variability drops below 10% for regions bigger than 68 mm^3^ for SRTM2-R1, 106 mm^3^ for LOGAN-DVR, and 300 mm^3^ for SRTM2-DVR, which are reasonable working sizes for the processing of multiple sclerosis lesions. The interhemispheric variability is also dependent upon the size of the regions analyzed. Whereas SUVR1-8 showed an intermediary reproducibility, SUVR0-2 for perfusion, or SUVR50-70 for tissue binding, showed poor reproducibility. These results suggest that [^11^C]PiB PET could be employed to assess simultaneously the variations in perfusion and myelin content in WM diseases associated with focal lesions.

Chen et al. [6] recently opened the perspective to quantify CBF with dynamic [^11^C]PiB PET by demonstrating a strong positive correlation between SRTM2-R1 and the relative delivery of [^15^O]H_2_O in 15 brain regions. Their study included many cortical areas and total WM, for which a positive correlation was found. In a longitudinal analysis, Bilgel et al. [47] corroborated the suitability of R1 derived from [^11^C]PiB PET as a reproducible surrogate for regional CBF in the GM but did not explore the WM. Finally, Heeman et al. conducted a test-retest assessment of SRTM2-R1 across 23 cortical regions [9] and showed a low variability of 1.70% in the global cortex, which reached a maximum of 5.78% in the gyrus rectus. Our study confirmed the reproducibility of SRTM2-R1 in the GM but extends the previous findings by showing an overall good reproducibility in the WM with an average of 1.74 ± 1.55% test-retest variability in the supratentorial WM that ranged from 1.88 to 2.56% in individual WM lobes. We further provide quantitative estimations of the influence of ROI sizes on test-retest variability: when large WM regions were considered, the reproducibility was overall good for most of the metrics investigated, whereas clear differences between metrics appeared when looking at smaller regions distributed in the WM. This highlights that smaller ROIs are more influenced by partial volume effects and yield less stable measurements [48, 49]. Consequently, in diseases such as MS, characterized by lesions with diverse sizes and volumes frequently below 10^4^ mm^3^, the variability of a PET-derived metric must not be taken as a constant but as a function of the volume of the regions investigated. Our analysis further points to the SRTM2-R1 as the most reliable method, out of those studied, for the quantification of CBF in small white matter lesions, with a variability below 10% for lesions exceeding 68 mm^3^, a threshold compatible with the analysis of most of the small WM lesions associated with MS or small vessel diseases.

In parallel to CBF, we explored three estimations of myelin content, the SRTM2-DVR, the LOGAN-DVR, and the SUVR50-70. Reinforcing the findings of Veronese et al. [12], who first investigated LOGAN-DVR in the same dataset, we showed that LOGAN-DVR was the most reproducible method for myelin quantification across the sizes of ROIs explored, reaching a test-retest variability below 10% for regions above 106 mm^3^. Meanwhile, the SRTM2-DVR, while being less reproducible with a test-retest variability below 10% for a region of 300 mm^3^ or more, differentiated better WM from GM than the LOGAN-DVR. Therefore, the loss in reproducibility might be overcome by the amplification of the image contrast and therefore not completely discredit the use of SRTM2-DVR, especially in cross-sectional studies. In addition, among the myelin content proxies, SRTM2-DVR showed the least correlations with SRTM2-R1. Indeed SRTM2 explicitly differentiates the influx rate from the binding potential expression while the LOGAN does not fully model it and has been shown to be penalized by bias especially when applied at voxel level [50]. By contrast, the poor reproducibility, and the lack of improvement for very large ROI obtained with SUVR50-70 demonstrate that this estimation of tissue binding in the WM is not appropriate for longitudinal studies.

Interestingly, in this population of adult subjects, brain CBF and myelin content showed inverse variations: regions with higher CBF displayed low indices of myelin content, and reciprocally, regions with low CBF were characterized by a higher myelin content. This finding is intriguing as increased blood flow and oxygen supply are required during developmental myelination to satisfy the high metabolic demand of myelinating oligodendrocytes [51, 52]. Therefore there is a great need for an imaging approach able to simultaneously investigate perfusion and myelin content dynamics in these diseases, a goal that may be reached by dynamic [^11^C]PiB PET.

Our study does not come without limitations. The number of subjects is limited and was not powered enough to investigate the relationship between CBF and aging, a factor that could contribute to WM diseases. As opposed to pioneer studies that have validated the use of [^11^C]PiB PET as a proxy of CBF, we did not further validate this approach through [^15^O]H_2_O PET or MRI with arterial spin labeling or dynamic contrast agent imaging, and future studies would be of great interest to assess the correlation between SRTM2-R1 derived from PET and MRI derived CBF measurements. Other kinetic models such as the multilinear reference tissue model 2 [53] could be of great interest to estimate both R1 and DVR, similarly to SRTM2, however this model has not yet been validated against PET with radiolabeled water for R1, and consequently has not been tested in this study. Here we did not sample arterial blood to assess the delivery rate of the tracer but instead relied on relative measures by using a reference region estimated using a supervised clustering algorithm [12, 53], which has the advantage of removing anatomical constraints for the determination of non-specific binding voxels and excludes voxels with a large blood component in their TAC [27]. Compared to previous studies that applied cerebellar GM as a reference region, our approach minimized the possible bias induced by voxel-wise heterogeneity in perfusion within the cerebellar cortex and opened the perspective to apply [^11^C]PiB PET to pathologies potentially affecting the cerebellar cortex. Using the SVCA instead of the cerebellar grey matter as a reference region did not produce different estimations of SRTM2-R1 nor did it modify our results in terms of ICC and WSCV. Finally, as scans used in this study were acquired on an HRRT PET, the relationship between reproducibility and ROI size may differ when other systems are used.

In conclusion, dynamic [^11^C]PiB PET is a promising multidimensional imaging tool that could provide simultaneous and reliable metrics for CBF and myelin content in the WM, opening the perspective to investigate the relationship between demyelination, remyelination, and local regulation of CBF in WM diseases. For longitudinal studies, SRTM2 modeling is the method of choice for simultaneous and independent CBF and myelin content estimates, while the analysis of small ROIs should consider the size dependence of test-retest variability. Future studies will decipher whether other fluorinated amyloid tracers with similar or improved affinity for WM myelin [13] may also display high lipophilicity, allowing their use as proxies of CBF [54].

## Conclusions

Our results indicate that [^11^C]PiB PET could be employed to assess jointly the variations in perfusion and myelin content in white matter diseases associated with focal lesions. In longitudinal studies SRTM2-R1 and DVR might be the optimal combination, yielding satisfying contrast and reliability, and SUVR0-2 and SUVR 50–70 should be avoided. The size and localization of the regions analyzed should be considered to capture significant changes for both metrics.

### Electronic supplementary material

Below is the link to the electronic supplementary material.

Supplementary Material 1

## Data Availability

The datasets generated during and/or analysed during the current study are available from the corresponding author on reasonable request.

## Abbreviations

ANTs: Advanced Normalization Tools
CBF: Cerebral Blood Flow
CPP: Comité de Protection des Personnes
DAC: Data Analysis Core
DVR: Distribution Volume Ration
FLAIR: Fluid-Attenuated Inversion Recovery
GM: Grey Matter
HRRT: High-Resolution Research Tomograph
ICC: Intraclass Correlation Coefficient
Logan-Ref: Logan graphical Reference method
MPRAGE: Magnetization-Prepared Rapid Gradient-Echo
MS: Multiple Sclerosis
ICM: Paris Brain Institute
%RD: percentage relative difference
PET: Positron Emission Tomography
R1: Relative Delivery
ROI: Region of Interest
SRTM2: Simplified Reference Tissue Model 2
SUVR: Standardized Uptake Value Ratio
TAC: Time Activity Curve
WM: White Matter
