# Supplementary material for: Simultaneous assessment of blood flow and myelin content in the brain white matter with dynamic [11 C]PiB PET: a test-retest study in healthy controls
Source: EJNMMI Res. 2024 May 27;14:50. doi: 10.1186/s13550-024-01107-4 (PMC11130116; doi:10.1186/s13550-024-01107-4)
Supplement: Supplementary file 1 — Supplementary Material 1 [file 13550_2024_1107_MOESM1_ESM.docx]

Paris, 10^th^ March 2024

Dear Dr Wim J. G. Oyen, Dear editors

Following the advice or reviewers we are happy to submit the revised version of our manuscript entitled “*Simultaneous assessment of blood flow and myelin content in the brain white matter with dynamic [^11^C]PiB PET: a test-retest study in healthy controls*” reference EJRE-D-23-00262, by Arya Yazdan Panah and colleagues to *European Journal of Nuclear Medicine and Molecular Imaging Research*.

We are very grateful to the Reviewers and Editors for their insightful comments and suggestions, which we feel have greatly improved our manuscript. We have addressed each comment of the reviewers in the response to Reviewers file and have modified our manuscript accordingly. Changes in the manuscript have been highlighted in red. We hope that this revised version of our paper will be considered for publication in *European Journal of Nuclear Medicine and Molecular Imaging Research*.

We guarantee that all co-authors have reviewed and approved the contents of the manuscript. We certify that the submission is not under review at any other publication. We have no conflict of interest to declare regarding this work.

We look forward to hearing soon from you.

Thank you for your consideration of this manuscript.

Sincerely,

Bruno Stankoff

Sorbonne Université

Institut du Cerveau - Paris Brain Institute

ICM, CNRS, Inserm, AP-HP

Hôpital de la Pitié Salpêtrière

F-75013, Paris, France

e-mail : [bruno.stankoff@aphp.fr](mailto:bruno.stankoff@aphp.fr); tel : 33157274465

**Reviewer #1:**

In this work, the authors examine the performance of dynamic [11C]PiB PET to quantify both cerebral blood flow (CBF) and myelin content simultaneously from a single PET examination, using either using different pharmacokinetic parameters from kinetic modelling, or alternatively by examining the standardized uptake value ratio (SUVR) from different periods of the acquisition. While none of these approaches are novel in-and-of themselves, this manuscript seeks to evaluate the application of this approach specifically to different areas of the white matter, as well as to small regions associated with focal lesions caused by multiple sclerosis (MS). Moreover, they evaluated the test-retest reliability of these outcomes and how sensitive these metrics are to region size. They conclude that [11C]PiB PET can fruitfully be used to assess both CBF and myelin content in white matter from a single examination with sufficiently good test-retest reliability, and that it can be applied to study MS lesions, provided they are not too small. Although there are no [15O]H2O PET examinations to compare the [11C]PiB-derived CBF outcomes to, and no MS patients to evaluate the validity of these outcomes directly, the study provides a valuable contribution nonetheless. Regarding the first point, there have already been other studies determining that these outcomes are correlated, and regarding the latter point, this is still a new field, and there have been other studies demonstrating the relationship between changes in PiB binding and focal lesions. I do not therefore see either of these as particularly important omissions. The paper is well-written and clear, and demonstrates coherent and useful results.

My comments, criticisms and suggestions are below:

**Reviewer’s comment:**

My primary criticism of the paper is with regard to the use of a supervised clustering algorithm (SCA) to extract a reference region. This might be a brilliant idea and improve the performance of the model, but its performance is not yet sufficiently validated. Although I am not an expert in PiB PET, nor in the use of the SCA, from reading some of the cited references, previous studies evaluating PiB PET as a marker of CBF seem to have made use of a cerebellar grey matter reference region. The SCA aims to select those voxels that best correspond to a reference tissue. However, this implies that the reference time activity curve will be derived from a different set of anatomical locations from each individual PET examination.

This complicates interpretation for two reasons.

Firstly, the SCA aims to define regions with low specific binding as a reference class, which is tailored to examining binding. However I am unsure how this would relate to R1: from the SRTM2 results, R1 and BPND do not appear to be correlated, and we know that some regions of the brain have more and less blood flow. When using an anatomically defined region such as cerebellar grey matter, relative blood flow is defined relative to this particular anatomical region, whose blood flow can reasonably be expected to exhibit certain properties by virtue of this region's physiology, and is interpretable as such. However, R1 relative to a set of voxels with low binding, but with an anatomical definition which varies between individuals, might complicate interpretation of this outcome, as different anatomical regions selected for each individual's brain might have different rates of blood flow to the reference region (i.e. K1') purely as a function of their different anatomical location.

Secondly, and related to the first point, the use of SCA might also complicate interpretation of the ICC. The ICC is a measure of the ability of an outcome to differentiate individuals. It does so by quantifying the proportion of the total variance (equal to the sum of true and error variance) in the data which is not attributable to error. Hence, the ICC can be increased either by diminishing the error variance (e.g. better quantification), or by artificially increasing the "true" variance. Because the ICC cannot differentiate individual-specific bias from true variance, if individuals have higher or lower estimates of true variance as a result of some source of individual-specific bias, then the model will interpret this as more true variance, and hence better performance according to the ICC. This is because individuals are being differentiated more successfully as a result of these biases: individuals with high values have high values at test and retest, and individuals with low values have low values at both test and retest; even if these high- and low-values are primarily because of this bias. With a reference region defined by SCA, it is plausible that the different reference regions introduce a source of individual-specific bias, which artificially exaggerates the ICC values. This could, for instance, be as a result of anatomical differences between individuals' brains resulting in better or worse performance of the SCA, or of different anatomical locations with different blood flow. I would appreciate it if the authors would either demonstrate that some subset of the results are similar when using a cerebellar grey matter reference region to make it clear that the SCA approach is not yielding dramatically different results, or otherwise demonstrate how the SCA does not give rise to these issues.

**Authors’ answer:**

We would like to thank the reviewer for this enlightening comment. In our work we decided to apply the supervised clustering algorithm because the cerebellum could not be used as a reference region for subjects with MS: indeed the neuroinflammation process in this disease can be diffuse and unpredictable, affecting also the cerebellum. The supervised clustering algorithm (SVCA) allowed the identification and extraction of reference region voxels, with no specific binding, both in the cortex and the cerebellum grey matter (CBGM). However, this supervised clustering method finally extracted voxels mainly in the cerebellum, resulting in an overlap of the reference regions derived from the SVCA algorithm or simply from the cerebellum grey matter.

Considering your comment, we have rerun our SRTM2 to quantify R1 with the cerebellar grey matter as a reference region to compare both methods (the maps and quantified results are shown below).

**SRTM2-R1 obtained with the cerebellar grey matter as the reference region.**

**SRTM2- R1 obtained with SVCA-based reference region.**

**Comparison of mean R1 in regions of interest with SVCA-based reference region (right) and CBGM (left)**

The figure above shows the different values for R1 in all regions for all subjects quantified with SVCA or cerebellar grey matter (CBGM) as reference region: note that whereas we observed a minor increase of R1 in some ROIs when quantifying with the cerebellum, the contrast between regions was preserved.

| ROI Name | ICC R1_svca | ICC R1_cbgm | WSCV R1_svca | WSCV R1_cbgm |
| --- | --- | --- | --- | --- |
| wm | 0.92 | 0.90 | 1.60E-02 | 1.67E-02 |
| cbgm | 0.75 | 0.75 | 1.55E-02 | 4.02E-03 |
| cbwm | 0.68 | 0.54 | 1.64E-02 | 1.34E-02 |
| cingulate_wm | 0.86 | 0.84 | 2.07E-02 | 1.92E-02 |
| frontal_wm | 0.87 | 0.88 | 1.87E-02 | 1.81E-02 |
| insula_wm | 0.87 | 0.94 | 2.08E-02 | 1.32E-02 |
| occipital_wm | 0.97 | 0.93 | 1.45E-02 | 2.07E-02 |
| parietal_wm | 0.91 | 0.83 | 2.05E-02 | 2.59E-02 |
| temporal_wm | 0.92 | 0.87 | 1.70E-02 | 2.05E-02 |

**Comparison of Intraclass correlation (ICC) and within-subject coefficient of variation (WSCV) of R1 obtained with SVCA-based or CBGM-based reference region.**

Whereas slight variations are detected, overall, the ICC and WSCV are not impacted by the method used (SVCA or CBGM).

**Mean R1 obtained using SCA and CBGM-based methods across all measures (test and retest) in brain regions of interest.**

| ROI | Pearson coefficient | P value |
| --- | --- | --- |
| Supratentorial WM | 0.848437 | 3.27E-05 |
| Cingulate WM | 0.841775 | 4.33E-05 |
| Frontal WM | 0.82012 | 1.00E-04 |
| Insula WM | 0.848426 | 3.27E-05 |
| Occipital WM | 0.932244 | 1.47E-07 |
| Parietal WM | 0.902882 | 1.68E-06 |
| Temporal WM | 0.865149 | 1.51E-05 |

**Pearson correlations between R1 were obtained using SVCA and CBGM-based methods across all measures (test and retest) in brain supratentorial white matter regions of interest. Results in grey matter are excluded as they are embedded in reference regions for both CBGM and SVCA.**

We found close and strong correlations for all supratentorial WM regions.

Regarding these results, we can conclude that no alarming differences emerge between those two quantification methods for R1 estimation in the white matter, the focus of our study.

To further address this comment in the main text, we have added the following sentences in the methods section (“PET Quantification” p.12l.2-4) and in the results section (page 23):

- Page 10, lines 5-7: “*To ensure that differences between subjects are not a result of reference region extraction we also use as a comparison point the cerebellar grey matter, widely used in the literature.*”
- Page 15, lines 10,11: “*SRTM2-R1 quantified using the SVCA and the cerebellar grey matter as reference regions were compared using ICC, WSCV and correlated using a Pearson correlation coefficient.”*
- Page 23, 24, lines 16-19, 1-4: “*Using the SVCA instead of the cerebellar grey matter as a reference region does not alter the results obtained. When quantifying SRTM2-R1 using both the SVCA and the cerebellar grey matter reference region, we found, in the WM, a respective ICC of 0.92 and 0.90, and a respective WSCV of 1.60E-02 and 1.67E-02. In all regions both metrics were close, and one quantification did not show inflation of these results compared to the other. In all supratentorial white matter regions, correlations between the two quantification methods were above 0.8.*
- Page 34, Lines 9-13: *“Using the SVCA instead of the cerebellar grey matter as a reference region did not produce different estimations of SRTM2-R1 nor did it modify our results in terms of ICC and WSCV.*

**Reviewer’s comment:**

While I am very aware that the percentage relative difference outcome is common in PET test-retest studies, it is worth remembering that it is not a particularly good outcome measure. As discussed in Baumgartner et al (DOI: 10.1186/s13550-018-0366-8), this outcome is insensitive to outliers owing to its individual-specific scaling. The within-subject coefficient of variation is a more statistically appropriate outcome because of its global scaling. This would not usually be of great relevance when evaluating a test-retest study since they are roughly equivalent. Still, since curve fitting is applied to these test-retest outcomes to assess volume sensitivity, it seems reasonable that the more appropriate outcome be examined. Owing to the near ubiquity of RD (%) in the PET literature though, both outcomes should probably be shown.

**Authors’ answer:**

We thank the reviewer for their suggestion. Following your recommendation and the article you are quoting, we are happy to include WSCV in this new version.

Modifications of the manuscript have been made to include WSCV, and in answer to the previous comment we also checked for the WXCV variations according to the reference region method used:

- Page 15, lines 6-9: “*(iii) The within-subject coefficient of variation (WSCV). As reported by Baumgartner et al [42] this metric has relevance in the case of PET test-retest studies as it performs global scaling instead of scaling at the individual level, therefore allowing to retain outliers if they exist.*”
- Page 27: Legend of Figure 4 has been modified adding the WSCV:
- Page 28, line 21: “*Overall the WSCV provided similar results compared to the %RD.*
- Page 29: Legend of Figure 5 has been modified adding the WSCV.
- Page 30, line 14: “*The interpretation of interhemispheric variability was not modified when WSCV was analyzed.*”

**Reviewer’s comment:**

The Logan-Ref model also requires the input of a k2' parameter as does the two-parameter SRTM2 model. While the derivation of k2' is described for SRTM2, I could not find a description of how it was determined for the Logan-Ref model.

**Authors’ answer:**

Using the same notation used for the SRTM equation in the manuscript, Logan-Ref can be written as (<https://doi.org/10.1097/00004647-199609000-00008>):

$$\frac{\int_{0}^{T} C_{T}(t)dt}{C_{T}(t)}=DVR\left[ \frac{\int_{0}^{T} C_{ref}(t)dt+C_{ref}(t)/k_{2}^{'}}{C_{T}(t)} \right]+int+\delta$$

According to the original paper, the term $C_{ref}(t)/k_{2}^{'}$ can be ignored if the ratio $\frac{C_{T}(t)}{C_{REF}(t)}$ is reasonably constant. As was shown by [(Veronese *et al.*, 2015)](https://www.zotero.org/google-docs/?v7vpdn), estimating the logan with or without the k2’ had minimal impact on the quantification.

The main results attesting this lack of impact from [Veronese *et al.*, 2015, PMID 26058700)](https://www.zotero.org/google-docs/?2kvfvT) were the following:

- DVR(Logan-k2) = 0.98 DVR(Logan) + 0.01, R2=0.99
- Absolute mean relative difference = 3±1%
- WM-GM contrast similar between the two methods

According to these reassuring results we decided to apply the same method as Veronese et al in this paper and further justified this choice in the method section:

- Page 12, lines 3-5: “*The k2' estimation was ignored during the Logan-Ref estimation as it was shown by Veronese et al [12] that estimating the Logan-Ref with and without k2 provided similar results.*”

**Reviewer’s comment:**

I was surprised by the lack of contrast between white and grey matter for the Logan-DVR. This would suggest to me that, even if this outcome is reliable, this implies poor face validity since we know that myelin content is higher in white matter. Why, then, does the conclusion suggest that Logan-DVR should be preferred for assessing myelin concentration: my reading was that SRTM2-DVR ought to be considered superior, despite its inferior reliability.

**Authors’ answer:**

We thank the reviewer for this comment and agree that our conclusion was not precise enough. We have now modified it as follows (fitting better with the abstract):

- Page 35, Lines 5-7: “*In longitudinal studies SRTM2-R1 and DVR might be the optimal combination, yielding satisfying contrast and reliability.”*

**Reviewer’s comment:**

Relatedly, my suspicion for why Logan-DVR fails to differentiate grey and white matter is that this might be caused by the selection of a t* time which is too early: this can cause this type of bias. I examined a previous article by this group using this tracer (DOI: 10.1007/s00259-019-04516-z), and 40 minutes was used as a t* time, rather than the 30 minutes used in this study. Might this perhaps improve the results?

**Authors’ answer:**

We thank the reviewer for pointing that out. Indeed, we tested different values for t* and finally selected the t* from the logan plot curve from the moment that it enters the linear regime. Following the reviewer’s recommendations, we have recalculated the Logan with a t* of 40 minutes and as shown in the comparison below, the contrast was not modified.

**Logan plot with fitting starting at t* = 40min and LOGAN-DVR of a subject**

**Logan plot with fitting starting at t* = 30min and LOGAN-DVR of a subject**

Both images are displayed with the same value range. As no changes were observed we decided to not make any modifications to the manuscript on that matter. Graphical methods such as the Logan plot are known to underestimate the DVR in the presence of noise. More in detail, the noise-induced underestimation in DVR images is dependent on the noise level and on the magnitude of the DVR, so the bias is more pronounced in regions with high DVR than regions with low DVR (<https://pubmed.ncbi.nlm.nih.gov/11138696/>). Our PET images were acquired on an HRRT system that, despite its higher resolution, is affected by a higher amount of noise compared to more modern PET cameras. We can speculate that the high noise of this system produced an underestimation of the DVR which was more pronounced in the white matter compared to the cortex (due to its higher DVR), thus reducing the contrast between the two regions. As the SRTM2 is less impacted by this effect, it should also be the preferred method therefore answering the reviewer’s remark on the t* selection.

**Reviewer’s comment:**

SRTM2 and the Logan-ref were used as kinetic models: SRTM2 is a nonlinear model which is slightly less susceptible to some of the biases of the linear models, but it is less robust to measurement error. Logan-ref, on the other hand, is a linear model which might be slightly more biased, but should be more robust to error. However, when selecting a linear model, Logan-ref seems like a peculiar choice when CBF is also of interest since it does not provide any information about CBF. MRTM2, on the other hand, is a linear model like Logan-ref, but it can be used to estimate both DVR and R1 provided that the tracer exhibits 1TC kinetics (which is also an assumption of SRTM and SRTM2). Moreover, with 1TC kinetics, there is no need for a t* value, which potentially resolves my prior concern about whether t* = 30 minutes is sufficiently late for the Logan-ref model. Might this be a useful model to include? Or is there a special reason for using the Logan-ref?

**Authors’ answer:**

The key reason for the choice of SRTM2 over MRTM2 was based on Chen et al., seminal study [(Chen *et al.*, 2015)](https://www.zotero.org/google-docs/?a1jiRG) in which they demonstrated a high correlation between SRTM2-R1 and relative K1 of radiolabeled water, the gold standard estimation of CBF. Having no access to such dual PET exams in our database, we would not be able to verify the same correlation with the MRTM2, and we acknowledge that we are not really mastering MRTM2 in our group. We therefore propose to remain consistent with the literature and use the same methodology. We take the point that MRTM2 is also an interesting model that could be of interest for further studies.

This has been added as a limitation of our study in the discussion:

- Pages 33, 34, Lines 20-21, 1-2: “*Other kinetic models such as the multilinear reference tissue model 2 [53] could be of great interest to estimate both R1 and DVR, similarly to SRTM2, however, this model has not yet been validated against PET with radiolabeled water for R1, and consequently has not been tested in this study*.”

**Reviewer’s comment:**

From Figures 4 and 5, I thought that fitting a model to the test-retest variability and reliability as a function of region size was an excellent idea, and it provides valuable conclusions regarding how these outcomes would be expected to perform when applied to small focal lesions. However, perhaps I missed it, but I did not see any description of what the function was that was fitted to this data, or whether any transformations were applied to the data to fit the model (e.g. was a Fisher's z-transformation applied to ICC values?).

**Authors’ answer:**

We thank the reviewer for their remark. No transformation was applied to the data. We tested polynomial and sigmoid curves to fit the data and concluded that the sigmoid provided the best fit by using the Akaike information criteria.

We have now modified it as follows (fitting better with the abstract):

- Page 28, Lines 3-5: “*No transformation was applied to the data. We tested polynomial and sigmoid curves to fit the data and concluded that the sigmoid provided the best fit by using the Akaike information criteria*.”
- Page 30, Lines 3-5: “*No transformation was applied to the data. We tested polynomial and sigmoid curves to fit the data and concluded that the sigmoid provided the best fit by using the Akaike information criteria.”*

**Minor comments**

Page 7 line 26: Missing reference

Reference Added (n°10)

Page 17 (and elsewhere): Mean and SD values are presented with two decimal places of precision. However, p-values such as 4.45e-15 are shown, implying an extraordinarily high level of precision. I think P < 0.001 would be more appropriate when the p values are low.

Corrected in tables.

Page 29 line 4: Bilgel et al is missing a citation.

Reference added.

This is more of a personal preference, so I leave this up to the preference of the authors. Although the word "reproducibility" is widely used for describing test-retest variability, it is also widely used to describe computational reproducibility of data analysis, i.e. the ability of computer code to re-generate the results of a given analysis. The word "repeatability" is more specific to test-retest measures and avoids this ambiguity. But if the authors prefer reproducibility, I will not complain.

We thank the reviewer for this remark and his/her comprehensiveness, and to avoid inconsistency and possible misses in the manuscript, we preferred if possible to keep the phrasing as is but take note of the comment for further studies.

This is not a criticism, but I wanted to commend the authors on the use of the repeated measures correlation: this is a great approach for analyzing this type of data. I have personally never encountered this metric before, but it solves a very common and pernicious problem, and I will most certainly keep it in mind for future studies!

We thank the reviewer for this positive comment.

**Reviewer #2:**

This is a well-written paper that addresses an interesting aspect of PET for white matter diseases. I have only a couple of suggestions and comments.

Abstract, line 24: The formulation "sensitivity of methods to spatial resolution" raises expectations that different spatial resolutions are studied. However, this part of the study is rather on the effects of the ROI size, including noise and partial volume effects. This sentence should be reformulated accordingly.

Thank you for the comment, we changed it accordingly in the abstract:

- Page 3 Line 13: “*The sensitivity of methods to the size of the region of interest was explored by simulating lesion masks obtained from 36 subjects with multiple sclerosis.*”

Introduction:

Page 6, line 36: Ref. 7 was published before ref. 6 and has therefore not used the method of ref. 6 subsequently.

Thank you for pointing out this error, reference 7 has been removed.

Page 7, line 21: Again, the "sensitivity to spatial resolution" is rather a sensitivity to ROI size.

Correction made.

Page 7, line 26: The reference is missing.

Correction made.

Materials and methods:

Page 7, line 49: The role of the 36 MS patients is not clear from this description. For which purpose are they included in the study?

These patients were included to define ROIs that reflect real MS lesions, both in shape, size, and brain location: this allowed to subsequently apply these masks on healthy subjects to study reproducibility in those locations.

This has been clarified in the materials and methods in the section:

- Page 8, lines 7-9: “*We also included 36 subjects with MS (21 women, age: 48±11 years), who were diagnosed according to the 2005 revised McDonald criteria [24]. These subjects allowed us to define masks of white matter lesions that* reflected real MS lesions, both in shape, size, and brain location, and that *could be used to study the effect of ROI size on the reproducibility of the metrics of interest.*”

Page 8, line 20: Which reconstruction software is used? Self-written or provided by vendor? Which version? Reference?

We thank the reviewer for the comment. We have added the software used and the corresponding reference in the Data Acquisition of the Materials & Methods section:

- Page 8, lines 17-19: *“Images were reconstructed using a three-dimensional ordinary Poisson-ordered subset expectation maximization algorithm with 10 iterations, with smoothing with a software developed by the HRRT users community [25].”*

Page 9, line 18: What are the kinetic classes?

The kinetic classes are a set of typical activities of the tracer in known regions that are averaged in a different group of subjects. These curves are typically expected behaviors for the region and are therefore useful to fit the data. Some precisions were added to the materials and methods (section “Data processing”) with a reference to the article where these classes were defined:

- Pages 9, 10, lines 18-19, 1,3: *“Kinetic classes are the standardized activity of the tracer in predefined regions of interests obtained from a reference population. The set of kinetic classes should represent all the possible ranges of tracer behavior in the brain. The kinetic classes used in this study are similar to those employed by Veronese et al. in their previous study [12], grey matter, white matter, and blood.”*

Page 9, line 32: Which software was used for SRTM2, Logan-Ref, and SUVR?

All PET modeling was done on self-written software in Matlab 2017. This was added to the materials and methods:

- Page 12, Lines 14,15: *“All PET modeling was written using MATLAB 2017, and the rest of the image processing pipelines were written in Python 3.7.”*

Page 11, line 50: There is a typo before "in each subject".

Correction made.

Page 13, line 25: %RD = 100*...

The 200 is correct as the relative difference is the absolute difference divided by the average of the 2 measures. The 2 is therefore coming from the average division. Consequently, no changes had to be made to the text.

Results:

Page 25, line 17: This is inconsistent with Fig. 4. An ICC that does not improve is shown in Fig. 4 for Binding and SUVR50-70.

We would like to thank the reviewer for taking the time to comment and for pointing out this mistake. This has been rectified in the text to be consistent with the information in Figure 4.

- Page 28, lines 19,20: “SUVR50-70 showed an ICC that did not improve even in larger lesion-like ROIs.”
